# The efficacy of radiofrequency ablation versus cryoablation in the treatment of single hepatocellular carcinoma: A population‐based study

**DOI:** 10.1002/cam4.3923

**Published:** 2021-05-07

**Authors:** Lei Chen, Yanqiao Ren, Tao Sun, Yanyan Cao, Liangliang Yan, Weihua Zhang, Tao Ouyang, Chuansheng Zheng

**Affiliations:** ^1^ Department of Radiology Union Hospital Tongji Medical College Huazhong University of Science and Technology Wuhan China; ^2^ Hubei Province Key Laboratory of Molecular Imaging Wuhan China; ^3^ Department of interventional radiology Union Hospital Tongji Medical College Huazhong University of Science and Technology Wuhan China

**Keywords:** cryoablation, efficacy, hepatocellular carcinoma, RFA, SEER

## Abstract

**Background:**

Radiofrequency ablation (RFA) is an effective treatment for single hepatocellular carcinoma (HCC), but it is difficult to use against tumors in some locations and often leads to incomplete ablation as a result of the heat‐sink effect. This study was conducted to evaluate the efficacy of cryoablation compared with that of RFA in the treatment of single HCC.

**Methods:**

This retrospective study was conducted based on the Surveillance, Epidemiology, and End Results (SEER) database. From 2004 to 2015, patients aged 40 to 79 diagnosed with HCC were included in the study. A propensity score matching (PSM) model was used to reduce selection biases.

**Results:**

Before PSM, the median overall survival (mOS) and median cancer‐specific survival (mCSS) in the RFA group were slightly longer than those in the cryoablation group (*p* > 0.05). In the subgroup analysis, the mOS and mCSS of patients with tumor sizes <3, 3–5, and >5 cm who received RFA treatment were longer than those of patients given cryoablation treatment, but there was no significant difference (*p* > 0.05). Similar results were presented in patients at American Joint Committee on Cancer (AJCC) stages I and II. After PSM, the mOS and mCSS were slightly better in the RFA group than the cryoablation group but without significant differences. Univariate and multivariate analysis showed that cryoablation treatment was not an unfavorable factor for OS and CSS before or after PSM (*p* > 0.05). In the multivariable competing risk model, non‐cancer‐specific death was taken as a competing factor and cryoablation was also not unfavorable for the survival of patients before and after PSM (*p* > 0.05).

**Conclusion:**

Cryoablation is non‐inferior to RFA therapy for single HCC patients without lymph node invasion or distant metastasis.

## Graphical abstract

This study compared the overall survival and cancer‐specific survival of patients with single HCC who received cryoablation and radiofrequency ablation. The results of this study showed that cryoablation had similar efficacy with radiofrequency ablation in the treatment of single HCC.

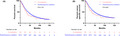

## 1 INTRODUCTION

Hepatocellular carcinoma (HCC) is the sixth most common cancer in the world and is the fourth leading cause of cancer‐related deaths.^1^ The incidence of HCC in the USA has increased in recent decades because of the increase in hepatitis C infections and non‐alcoholic cirrhosis.^2^ For early HCC, the European Association for the Study of the Liver recommends the use of transplantation, liver resection, or ablation as first‐line treatments.^3^ However, most patients with early HCC are not suitable candidates for transplantation because of a lack of available organs and Milan criteria restrictions. Liver resection can prolong the survival times of patients, but the associated complications are complex, and the 5‐year tumor recurrence rate with this treatment can reach 70%.^4^ Thus, minimally invasive techniques that can destroy tumors are more widely used. Radiofrequency ablation (RFA) is the most commonly used ablation technique and it can achieve a similar effect to liver resection in the treatment of HCC (with tumor diameters of less than 2 cm).^5^, ^6^ For tumors less than 3 cm in diameter, the 3‐year overall survival rates of HCC patients range from 67% to 84%.^7^ However, in the treatment of larger tumors, RFA often results in incomplete ablation because of the heat‐sink effect (especially for tumors located near major blood vessels), resulting in increased tumor recurrence.

Recently, cryoablation has been more frequently used to treat solid tumors and has shown good efficacy.^8^, ^9^, ^10^, ^11^ It can destroy tumor cells by forming intracellular ice and extracellular crystals, which damage cell membranes and intracellular organelles, leading to tumor cell death. Several studies have shown that cryoablation can destroy tumor cells and induce the release of their intracellular contents into the extracellular space, which can trigger the immune response, killing residual tumor cells and reducing tumor recurrence.^12^, ^13^ During cryoablation, an ice ball forms around the tip of the probe within tumor tissue, and the ice can be precisely and intraprocedurally monitored via various imaging techniques such as ultrasound, magnetic resonance imaging, or computed tomography, facilitating more precise tumor treatment.

Nowadays, some scholars believe that patients with single HCC tumors that neither invade vessels nor metastasize to distant organs and have preserved liver function should be defined as early stage and be given radical treatment.^3^, ^14^, ^15^, ^16^ Interest in applying cryoablation to the treatment of HCC is growing. However, it is unclear whether patients with single HCC tumors will benefit more from cryoablation or RFA. Therefore, we conducted a population‐based study to compare the efficacy of cryoablation versus RFA in the treatment of single HCC tumors.

## 2 METHODS AND MATERIALS

### 2.1 Patients

The data used in this study were extracted from the Surveillance, Epidemiology, and End Results (SEER)‐18 registries database, which includes approximately 28% of the population of the United States. The college ethics committee approved the retrospective study. The requirement to obtain informed consent was waived by the institutional review board, as it was a population‐based study.

The following inclusion criteria were applied: (a) patients diagnosed with HCC (ICD‐O‐3, code 8170/3, 8171/3, 8172/3, 8173/3, 8174/3, 8175/3, and site code C220) from 2004 to 2015; (b) patients aged between 40 and 79; (c) patients with single tumors that did not invade lymph nodes or metastasize to distant organs; (d) single tumor patients with regional invasion.

Patients with American Joint Committee on Cancer (AJCC) stages N1 or NX and M1 or MX were excluded. Patients with a survival month code of 0 were excluded because this indicated that the patient was lost to follow‐up after diagnosis. Finally, 3614 patients were enrolled, of which 104 were treated with cryoablation and 3510 were treated with RFA (Figure S1).

### 2.2 Outcomes and statistical analysis

The endpoints of the study were overall survival (OS) and cancer special survival (CSS). OS was defined as the interval from the diagnosis of HCC to the death of the patient. CSS was defined as the interval from the diagnosis of HCC to death caused by HCC of the patient.

The data were extracted using SEER*Sat software (version 8.3.6). All continuous variables were transformed into categorical variables. Chi‐squared test or Fisher's exact test were used to compare the differences in the baseline characteristics and the characteristics after PSM between the RFA group and cryoablation group. The OS and CSS were plotted on Kaplan–Meier curves and compared with the log‐rank test. The Cox proportional hazard model was used to analyze the predictors for OS and CSS before and after PSM. Variables with *p*‐values of less than 0.1 in univariate analysis were included in multivariate analysis. To compete the risk analysis, non‐cancer‐specific death was taken as the competing factor, and the Gray's test was used to identify statistical differences between the two groups. The Fine–Gray multivariable regression model was used to identify factors associated with the risk of survival of patients treated with RFA or cryoablation.

To reduce selection bias and the influence of potential confounding factors, PSM was used to generate better matching groups. The characteristics of age, gender, tumor stage, AJCC stage, size, marital status, and chemotherapy treatment were included in the PSM analysis. The optimal caliper was set as 0.1 and 498 patients were matched by the one to five nearest neighbor approach. Among these patients, 104 received cryoablation treatment and 394 received RFA treatment. *p*‐values < 0.05 were considered statistically significant, except for in the univariate analysis and all statistical analyses were two‐tailed. Analyses were conducted with SPSS (version 24.0) and Stata (version 14.0).

## 3 RESULTS

### 3.1 Baseline characteristics

A total of 3614 patients were included in the study, of which 2769 patients were male, and 845 patients were female. Among them, 104 patients received cryoablation treatment and 3510 patients received RFA treatment. Before PSM, the patients in the cryoablation group were older than those in the RFA group (*p* = 0.003), the year of diagnosis in the cryoablation group was earlier than that in the RFA group (*p* = 0.002), and there were more white patients in the cryoablation group than the RFA group (*p* = 0.02) (Table 1). After PSM, there were no significant differences in the characteristics between the two groups (All *p* > 0.05) (Table 1).

**TABLE 1:** Characteristics of patients between the two groups before and after PSM

	Before PSM			After PSM		
Characteristics	RFA group (3510 patients, %)	Cryoablation group (104 patients, %)	*p* value	RFA group (394 patients, %)	Cryoablation group (104 patients, %)	*p* value
Age (Years)			0.003			0.587
40–49	231 (6.6)	4 (3.8)		10 (2.5)	4 (3.8)	
50–59	1301 (37.1)	35 (33.7)		139 (35.3)	35 (33.7)	
60–69	1303 (37.1)	30 (28.8)		133 (33.8)	30 (28.8)	
70–79	675 (19.2)	35 (33.7)		112 (28.4)	35 (33.7)	
Gender			0.692			0.388
Male	2691 (76.7)	78 (75.0)		311 (78.9)	78 (75.0)	
Female	819 (23.3)	26 (25.0)		83 (21.1)	26 (25.0)	
Year of diagnosis			0.002			0.415
2004–2007	760 (21.6)	35 (33.7)		107 (27.2)	35 (33.7)	
2008–2011	1059 (30.2)	36 (34.6)		145 (36.8)	36 (34.6)	
2012–2015	1691 (48.2)	33 (33.7)		142 (36)	33 (33.7)	
Tumor stage			0.599			0.273
Localized	2942 (83.8)	86 (82.7)		347 (88.1)	86 (82.7)	
Regional	535 (15.2)	16 (15.4)		44 (11.2)	16 (15.4)	
Unknown/Unstaged	33 (1.0)	2 (1.9)		3 (0.7)	2 (1.9)	
AJCC stage			0.797			0.360
I	2318 (66.0)	64 (61.5)		277 (70.3)	64 (61.5)	
II	941 (26.8	32 (30.8)		97 (24.6)	32 (30.8)	
III	165 (4.7)	6 (5.8)		14 (3.6)	6 (5.8)	
UNK stage	86 (2.5)	2 (1.9)		6 (1.5)	2 (1.9)	
Tumor size (cm)			0.283			0.607
No more than 3	2068 (58.9)	60 (57.7)		234 (59.4)	60 (57.7)	
3–5	971 (27.7)	26 (25.0)		111 (28.2)	26 (25.0)	
Larger than 5	291 (8.3)	14 (13.5)		37 (9.4)	14 (13.5)	
Unknown	180 (5.1)	4 (3.8)		12 (3)	4 (3.8)	
Ethnicity			0.020			0.904
White	2335 (66.5)	81 (77.9)		311 (78.9)	81 (77.9)	
Black	414 (11.8)	12 (11.5)		47 (11.9)	12 (11.5)	
Other	761 (21.7)	11 (10.6)		36 (9.2)	11 (10.6)	
Marital status			0.392			0.482
Married	1872 (53.3)	52 (50.0)		201 (51)	52 (50.0)	
Unmarried	1502 (42.8)	50 (48.1)		176 (44.7)	50 (48.1)	
Unknown	136 (3.9)	2 (1.9)		17 (4.3)	2 (1.9)	
Chemotherapy			0.707			0.708
Yes	1120 (31.9)	35 (33.7)		125 (31.7)	35 (33.7)	
No	2390 (60.1)	69 (66.3)		269 (68.3)	69 (66.3)	

### 3.2 Efficacy

**FIGURE 1:**
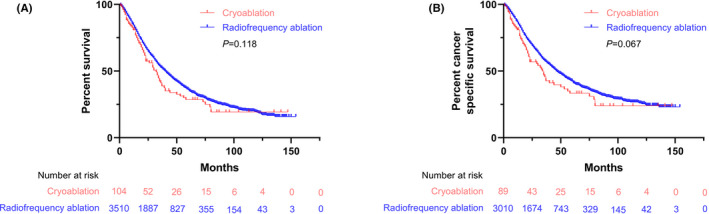
Kaplan–Meier curve of overall survival (A) and cancer‐specific survival (B) of patients with cryoablation and radiofrequency ablation (RFA) before PSM

Before PSM, there was no significant difference in the median OS (mOS) between the cryoablation group (40 months, 95%CI: 37.8, 42.2) and the RFA group (32 months, 95%CI: 25.1, 38.9; *p* = 0.118) (Figure 1A), and similar results were seen in the median CSS (mCSS) between the cryoablation (47 months, 95%CI: 43.9, 50.1) and RFA groups (34 months, 95%CI: 27.2, 40.8; *p* = 0.067) (Figure 1B). From the subgroup analysis, the mOS and mCSS of patients with tumor diameters of no more than 3 cm were longer in the cryoablation group (51 months, 95%CI: 47.5, 54.5; 61 months, 95%CI: 55.1, 66.9, respectively) than the RFA group (40 months, 95%CI: 28.8, 51.2; 56 months, 95%CI: 16.1, 95.9, respectively), but there were no significant differences between the two groups (*p* = 0.588 and *p* = 0.405, respectively) (Figure S2A and B). In the subgroup of patients with single tumors of no more than 5 cm, the mOS and mCSS in the RFA group (43 months, 95%CI: 41.2, 44.8; 51 months, 95%CI: 47.2, 54.8, respectively) and the cryoablation group (34 months, 95%CI: 24.5, 43.5; 37 months, 95%CI: 11.8, 62.2, respectively) were similar (*p* = 0.557 and *p* = 0.393, respectively) (Figure S2C and D). Similar results were found for the patients with tumors larger than 5 cm: the mOS and mCSS in the RFA group (22 months, 95%CI: 18.2, 25.8; 23 months, 95%CI: 18.1, 27.9, respectively) were similar to those in the cryoablation group (26 months, 95%CI: 0, 51.2; 26 months, 95%CI: 0, 48.8, respectively) (*p* = 0.158 and *p* = 0.185, respectively) (Figure S2E and F). For AJCC stages I and II, the mOS and mCSS in the RFA group (42 months, 95%CI: 39.5, 44.5; 50 months, 95%CI: 46.4, 53.6, respectively) were similar to those in the cryoablation group (34 months, 95%CI: 27.2, 40.8; 36 months, 95%CI: 26.9, 45.1, respectively) (*p* = 0.160 and *p* = 0.084, respectively) (Figure S2G and H).

**FIGURE 2:**
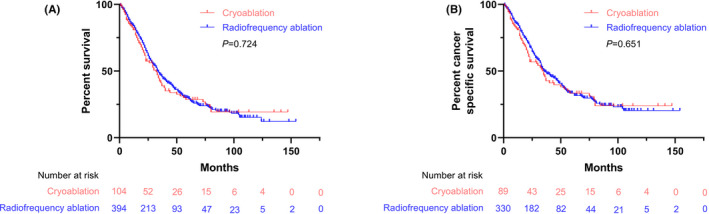
Kaplan–Meier curve of overall survival (A) and cancer‐specific survival (B) of patients with cryoablation and radiofrequency ablation (RFA) after PSM

After PSM, the mOS and mCSS in the two groups were compared, and the results in the RFA group (33 months, 95%CI: 28.8, 37.2; 36 months, 95%CI: 29.9, 42.1, respectively) were similar to those in the cryoablation group (32 months, 95%CI: 25.1, 38.9; 34 months, 95%CI: 27.2, 40.8, respectively) (*p* = 0.724 and *p* = 0.651, respectively) (Figure 2A and B).

### 3.3 Predictors for OS and CSS

The univariate analysis showed that the RFA was not an independent favorable factor for OS (HR: 0.830; 95%CI: 0.656, 1.051, *p* = 0.121) (Table 2). However, multivariate analysis showed that RFA was not an independent favorable factor for CSS (HR: 0.940; 95%CI: 0.719, 1.227, *p* = 0.648) before PSM (Table 3). After PSM, similar results were obtained and RFA was not an independent favorable factor for OS (HR: 0.955; 95%CI: 0.735, 1.239, *p* = 0.727) (Table 4) or CSS (HR: 0.935; 95%CI: 0.696, 1.256, *p* = 0.654) (Table 5). In the multivariable competing risk model analysis before PSM, patients with RFA did not show superior survival to patients given cryoablation (HR: 0.887, 95%CI: 0.681–1.155, *p* = 0.374) (Table S1). After PSM, univariable competing risk model analysis showed that patients provided RFA showed no superior survival to patients treated with cryoablation (HR: 0.843, 95%CI: 0.643–1.105, *p* = 0.215) (Table S2).

**TABLE 2:** Predictors for overall survival before PSM

	Univariate analysis		Multivariate analysis	
Characteristics	HR (95%CI)	*p*	HR (95%CI)	*p*
Age (Years)				
40–49	Reference		Reference	
50–59	1.190 (0.987,1.434)	0.068	1.165 (0.966,1.405)	0.110
60–69	1.175 (0.974,1.418)	0.092	1.252 (1.036,1.513)	0.020
70–79	1.520 (1.251,1.847)	<0.001	1.568 (1.288,1.908)	<0.001
Gender				
Male	Reference			
Female	0.928 (0.837,1.029)	0.159		
Year of diagnosis				
2004–2007	Reference		Reference	
2008–2011	0.754 (0.679,0.837)	<0.001	0.795 (0.714,0.884)	<0.001
2012–2015	0.650 (0.580,0.728)	<0.001	0.686 (0.611,0.770)	<0.001
Tumor stage				
Localized	Reference		Reference	
Regional	1.598 (1.428,1.789)	<0.001	1.283 (1.119,1.471)	<0.001
Unknown/Unstaged	1.668 (1.140,2.440)	<0.001	1.220 (0.732,1.032)	0.446
AJCC stage				
I	Reference		Reference	
II	1.296 (1.175,1.429)	<0.001	1.174 (1.052,1.313)	0.005
III	2.404 (2.015,2.869)	<0.001	1.528 (1.215,1.922)	<0.001
UNK stage	1.700 (1.175,1.429)	<0.001	1.256 (0.846,1.866)	0.258
Tumor size (cm)				
No more than 3	Reference		Reference	
3–5	1.586 (1.439,1.748)	<0.001	1.483 (1.343,1.637)	<0.001
Larger than 5	2.048 (1.779,2.356)	<0.001	1.640 (1.385,1.942)	<0.001
Unknown	1.511 (1.255,1.819)	<0.001	1.312 (1.043,1.651)	0.020
Ethnicity				
White	Reference		Reference	
Black	0.969 (0.846,1.110)	0.649	0.993 (0.866,1.138)	0.918
Other	0.690 (0.616,0.772)	<0.001	0.681 (0.607,0.763)	<0.001
Marital status				
Married	Reference		Reference	
Unmarried	1.414 (1.045,1.246)	0.003	1.173 (1.072,1.284)	<0.001
Unknown	0.912 (0.714,1.165)	0.462	0.963 (0.753,1.231)	0.762
Chemotherapy				
Yes	Reference			
No	0.970 (0.885,1.065)	0.525		
Treatment				
Cryoablation	Reference			
RFA	0.830 (0.656,1.051)	0.121		

**TABLE 3:** Predictors for cancer‐specific survival before PSM

	Univariate analysis		Multivariate analysis	
Characteristics	HR (95%CI)	*p*	HR (95%CI)	*p*
Age (Years)				
40–49	Reference		Reference	
50–59	1.221 (0.978,1.526)	0.078	1.189 (0.951,1.487)	0.128
60–69	1.239 (0.990,1.549)	0.061	1.339 (1.069,1.677)	0.011
70–79	1.699 (1.348,2.140)	<0.001	1.739 (1.377,2.196)	<0.001
Gender				
Male	Reference			
Female	0.938 (0.833,1.057)	0.295		
Year of diagnosis				
2004–2007	Reference		Reference	
2008–2011	0.700 (0.622,0.789)	<0.001	0.758 (0.671,0.856)	<0.001
2012–2015	0.526 (0.461,0.599)	<0.001	0.564 (0.493,0.646)	<0.001
Tumor stage				
Localized	Reference		Reference	
Regional	1.743 (1.535,1.979)	<0.001	1.326 (1.135,1.549)	<0.001
Unknown/Unstaged	1.693 (1.100,2.606)	0.017	1.019 (0.578,1.795)	0.948
AJCC stage				
I	Reference		Reference	
II	1.382 (1.235,1.546)	<0.001	1.240 (1.091,1.409)	0.001
III	2.805 (2.316,3.397)	<0.001	1.621 (1.255,2.094)	<0.001
UNK stage	1.878 (1.424,2.478)	<0.001	1.409 (0.913,2.175)	0.121
Tumor size (cm)				
No more than 3	Reference		Reference	
3–5	1.790 (1.600,2.003)	<0.001	1.639 (1.462,1.837)	<0.001
Larger than 5	2.409 (2.058,2.819)	<0.001	1.808 (1.491,2.192)	<0.001
Unknown	1.720 (1.394,2.121)	<0.001	1.436 (1.101,1.872)	0.007
Ethnicity				
White	Reference		Reference	
Black	0.948 (0.810,1.110)	0.507	0.978 (0.834,1.146)	0.780
Other	0.681 (0.599,0.776)	<0.001	0.669 (0.586,0.764)	<0.001
Marital status				
Married	Reference		Reference	
Unmarried	1.154 (1.043,1.278)	0.006	1.202 (1.083,1.333)	0.001
Unknown	0.910 (0.678,1.221)	0.530	1.010 (0.751,1.357)	0.948
Chemotherapy				
Yes	Reference			
No	0.946 (0.851,1.052)	0.307		
Treatment				
RFA	Reference		Reference	
Cryoablation	0.783 (0.601,1.020)	0.069	0.940 (0.719,1.227)	0.648

**TABLE 4:** Predictors for overall survival after PSM

Characteristics	Univariate analysis		Multivariate analysis	
	HR (95%CI)	*p*	HR (95%CI)	*p*
Age (Years)				
40–49	Reference			
50–59	1.016 (0.515,2.004)	0.964		
60–69	1.060 (0.535,2.100)	0.868		
70–79	1.295 (0.655,2.560)	0.458		
Gender				
Male	Reference			
Female	1.098 (0.849,1.420)	0.477		
Year of diagnosis				
2004–2007	Reference		Reference	
2008–2011	0.886 (0.694,1.131)	0.331	0.867 (0.671,1.121)	0.276
2012–2015	0.686 (0.508,0.927)	0.014	0.696 (0.514,0.942)	0.019
Tumor stage				
Localized	Reference		Reference	
Regional	1.447 (1.059,1.976)	0.020	1.283 (0.871,1.888)	0.207
Unknown/Unstaged	1.694 (0.542,5.291)	0.364	2.093 (0.374,11.701)	0.400
AJCC stage				
I	Reference		Reference	
II	0.911 (0.706,1.176)	0.473	0.907 (0.683,1.204)	0.499
III	3.505 (2.184,5.624)	<0.001	2.166 (1.129,4.157)	0.020
UNK stage	1.880 (0.835,4.231)	0.127	0.971 (0.269,3.499)	0.964
Tumor size (cm)				
No more than 3	Reference		Reference	
3–5	1.653 (1.295,2.111)	<0.001	1.688 (1.317,2.163)	<0.001
Larger than 5	2.371 (1.693,3.320)	<0.001	1.768 (1.150,2.718)	0.009
Unknown	2.342 (1.401,3.914)	0.001	2.098 (1.178,3.738)	0.012
Ethnicity				
White	Reference			
Black	1.032 (0.725,1.471)	0.860		
Other	0.749 (0.504,1.114)	0.154		
Marital status				
Married	Reference			
Unmarried	1.131 (0.910,1.407)	0.267		
Unknown	0.587 (0.300,1.150)	0.121		
Chemotherapy				
Yes	Reference			
No	1.166 (0.918,1.480)	0.208		
Treatment				
Cryoablation	Reference			
RFA	0.955 (0.735,1.239)	0.727		

**TABLE 5:** Predictors for cancer‐specific survival after PSM

	Univariate analysis		Multivariate analysis	
Characteristics	HR (95%CI)	*p*	HR (95%CI)	*p*
Age (Years)				
40–49	Reference			
50–59	1.196 (0.522,2.740)	0.673		
60–69	1.269 (0.551,2.919)	0.576		
70–79	1.658 (0.723,3.801)	0.232		
Gender				
Male	Reference			
Female	1.141 (0.850,1.533)	0.380		
Year of diagnosis				
2004–2007	Reference		Reference	
2008–2011	0.786 (0.595,1.039)	0.091	0.749 (0.558,1.004)	0.053
2012–2015	0.579 (0.413,0.812)	0.002	0581 (0.414,0.816)	0.002
Tumor stage				
Localized	Reference		Reference	
Regional	1.603 (1.130,2.272)	0.008	1.475 (0.939,2.315)	0.091
Unknown/Unstaged	1.360 (0.338,5.479)	0.665	0.941 (0.118,7.482)	0.954
AJCC stage				
I	Reference		Reference	
II	0.978 (0.735,1.303)	0.881	0.909 (0657,1.258)	0.565
III	3.717 (2.214,6.242)	<0.001	1.946 (0.944,4.015)	0.071
UNK stage	2.139 (0.793,5.775)	0.133	1.846 (0.392,8.694)	0.438
Tumor size (cm)				
No more than 3	Reference		Reference	
3–5	1.751 (1.324,2.315)	<0.001	1.849 (1.390,2.459)	<0.001
Larger than 5	2.651 (1.824,3.852)	<0.001	2.009 (1.255,3.215)	0.004
Unknown	4.627 (2.539,8.431)	<0.001	3.764 (1.929,7.346)	<0.001
Ethnicity				
White	Reference			
Black	1.001 (0.664,1.507)	0.997		
Other	0.755 (0.472,1.208)	0.241		
Marital status				
Married	Reference			
Unmarried	1.113 (0.868,1.427)	0.400		
Unknown	0.561 (0.230,1.372)	0.205		
Chemotherapy				
Yes	Reference			
No	1.200 (0.917,1.572)	0.185		
Treatment				
Cryoablation	Reference			
RFA	0.935 (0.696,1.256)	0.654		

## 4 DISCUSSION

A hepatectomy is recommended for patients with single HCC without invasion of vessels, lymph nodes, or metastasis to distant organs. However, some patients cannot receive a resection because of their poor physical condition, high‐risk status, or vulnerability to complications.^17^, ^18^ Minimally invasive treatments are used for these patients and can achieve satisfactory efficacies, especially RFA.^19^ Substantial evidence shows that patients with single HCC receive survival benefits from RFA alone or in combination with other treatments.^20^, ^21^ However, the disadvantage of using RFA is that it is difficult to apply when the HCC is located under the diaphragm or is adjacent to other important organs. In these cases, cryoablation is recommended, but insufficient data limit the application of cryoablation in the treatment of HCC. In a high‐quality study comparing the efficacies of cryoablation and RFA in the treatment of HCC patients with cirrhosis, tumor diameters of ≤4 cm and no more than two tumors, the rates of local tumor progression at 1, 2, and 3 years were higher in the cryoablation group than the RFA group (*p* = 0.041); however, there were no significant differences in the overall survival rates at 1, 3, or 5 years between the two groups (*p* = 0.747).^22^ Although, previous studies have shown that patients with unresectable liver cancer, local HCC, tumors larger than 2 cm and one or two tumors of no more than 4 cm who received cryoablation had similar survival benefits compared with those who received RFA.^22^, ^23^, ^24^, ^25^ The EASL guidelines classify patients with HCC more finely and recommends different treatments for different HCC patients. However, no studies have compared the efficacy of cryoablation with RFA for patients with single HCC not invading lymph nodes or metastasizing to distant organs and especially those patients with single HCC, early stage cancer, or with different tumor sizes. Thus, we conducted the current study to compare the efficacies of cryoablation and RFA in the treatment of a single HCC using the SEER database.

Previous studies have shown HCC patients to have an OS range of 8 to 29 months when treated with cryoablation alone or in combination with other therapies.^24^, ^26^, ^27^ The mOS in this study was longer than that in the previous study, mainly because the previous study recruited patients with larger tumor diameters and more tumors. Although, patients with single HCC were included in this study, the efficacy of cryoablation was found to be similar to that of RFA, which is consistent with previous studies that recruited patients with different stages of HCC and compared cryoablation with RFA efficacies. Prospective and retrospective studies have shown that patients with small HCC tumors received more survival benefits compared with those provided other treatments. Therefore, in this study, patients were divided into four subgroups according to tumor size and AJCC stage. The results of this study indicate that patients received RFA and cryoablation had similar mOS and mCSS as patients with tumors <3, 3–5, and >5 cm in diameter, as well as patients at AJCC stages I and II. RFA alone was not recommended as a first‐line treatment for patients with single tumors larger than 5 cm, although, the results of cryoablation and RFA were similar in this study. Many studies have shown that patients with HCC tumors of >5 cm receive more survival benefits from RFA combined with other treatments.^21^, ^28^ Thus, for patients with single HCC tumors of >5 cm in diameter, the efficacy of cryoablation combined with other treatments compared with that of RFA combined with other treatments remains to be further evaluated. After reducing the influence of the patients’ baseline characteristics via PSM, the efficacy of cryoablation was found to be similar to that of RFA. Combined with the results of previous studies, our findings show that cryoablation may be more widely used in the treatment of HCC and has good efficacy.

Previous research has shown that tumor burden and liver function are independent predictors for patients with HCC who receive radical treatment (resection or ablation).^29^, ^30^ In this study, the patients’ liver function was not included in the Cox proportional risk model, as the SEER database does not contain this information; however, patients’ age, gender, year of diagnosis, tumor stage, AJCC stage, tumor size, ethnicity, marital status, chemotherapy, and treatment were included in the model. Prior to PSM, the patient's age, year of diagnosis, tumor stage, AJCC stage, tumor size, ethnicity, and marital status were related to the patient's OS and CSS. After PSM, only the year of diagnosis, AJCC stage, and tumor size were associated with the OS and CSS. The results suggest that patients diagnosed with HCC earlier and with higher AJCC stages and larger tumor sizes have poorer prognoses, and these results are consistent with the findings of other population‐based studies. Patients diagnosed with HCC earlier may have had poorer survival outcomes because they received less advanced surgical techniques and/or treatment methods. Patients older than 60 years had a higher risk of comorbidities than younger patients. Thus, non‐cancer‐specific death was taken as a competing factor for comparing the efficacies between the RFA and cryoablation groups. However, cryoablation still did not show an increased mortality risk compared with RFA, indicating that patients with single HCCs receive similar survival benefits from cryoablation as from RFA. Although, the findings of the current study might provide evidence for clinicians when choosing the most suitable treatments for patients with single HCC who are not candidates for RFA, there are many unresolved problems in the cryoablation treatment of patients with HCC. Recently, studies have shown that HCC patients with single tumors up to 3 cm who receive RFA could experience similar survival benefits compared with those who receive liver resection, which led to guidelines recommending RFA as the first‐line treatment for patients with HCC of no more than 3 cm.^3^, ^19^, ^20^ However, whether similar survival benefits are bestowed on patients receiving cryoablation as patients receiving liver resection is still unknown and has not been the focus of any previous studies. Thus, future studies should aim to include these patients.

This study had some limitations. First, this was a retrospective study, which inevitably produces selective bias, although, we used PSM to reduce this. Second, this was a population‐based study, the central pathological review and the data on earlier tumor control endpoints, such as progression‐free survival and time to progression, as well as information about complications, could not be obtained and evaluated, which may affect the accuracy of the results. However, previous studies have shown no statistical differences in the incidences of complications in patients that underwent cryoablation compared with those provided RFA. Nevertheless, a prospective randomized trial is needed to confirm the results of this study.

## 5 CONCLUSION

This population‐based study demonstrated that treatment with cryoablation provides similar survival benefits to patients with single HCC tumors treatment compared with RFA. Therefore, if patients with single HCCs are unsuitable for RFA or unwilling to receive RFA, cryoablation may be a selective choice.

## ETHICAL APPROVAL AND CONSENT TO PARTICIPATE

The research do not need to be reviewed by the ethics committee because the data were from SEER database and the written informed consent was exempted. However, the data used in the research was permitted by the SEER database management department.

## CONFLICT OF INTEREST

All authors declare there is no conflict of interest.

## Supporting information

Supplementary Material

## Data Availability

The data could be found in SEER database (https://seer.cancer.gov/data/).
