# Supplementary material for: The efficacy of radiofrequency ablation versus cryoablation in the treatment of single hepatocellular carcinoma: A population‐based study
Source: Cancer Med. 2021 May 7;10(11):3715–25. doi: 10.1002/cam4.3923 (PMC8178489; doi:10.1002/cam4.3923)

**Supplementary materials:**

**Table 1: Predictors for competing risk before PSM**

| Characteristics | Univariate analysis |  | Multivariate analysis |  |
| --- | --- | --- | --- | --- |
|  | HR (95%CI) | P | HR (95%CI) | P |
| **Age (Years)** |  |  |  |  |
| 40-49 | Reference |  | Reference |  |
| 50-59 | 1.214 (0.971,1.519) | 0.089 | 1.219 (0.971,1.531) | 0.088 |
| 60-69 | 1.247 (0.996,1.562) | 0.055 | 1.383 (1.100,1.738) | 0.006 |
| 70-79 | 1.614 (1.281,2.033) | <0.001 | 1.669 (1.318,2.113) | <0.001 |
| **Gender** |  |  |  |  |
| Male | Reference |  |  |  |
| Female | 0.96 (0.853,1.081) | 0.502 |  |  |
| **Year of diagnosis** |  |  |  |  |
| 2004-2007 | Reference |  | Reference |  |
| 2008-2011 | 0.754 (0.672,0.846) | <0.001 | 0.807 (0.716,0.910) | <0.001 |
| 2012-2015 | 0.558 (0.492,0.633) | <0.001 | 0.605 (0.530,0.690) | <0.001 |
| **Tumor stage** |  |  |  |  |
| Localized | Reference |  | Reference |  |
| Regional | 0.611 (0.537,0.696) | <0.001 | 0.810 (0.690,0.950) | 0.010 |
| Unknown/Unstaged | 0.969 (0.635,1.48) | 0.885 | 0.857 (0.494,1.487) | 0.584 |
| **AJCC stage** |  |  |  |  |
| I | Reference |  | Reference |  |
| II | 1.371 (1.225,1.534) | <0.001 | 1.268 (1.112,1.446) | <0.001 |
| III | 2.703 (2.195,3.329) | <0.001 | 1.759 (1.339,2.311) | <0.001 |
| UNK stage | 1.869 (1.419,2.461) | <0.001 | 1.474 (0.939,2.316) | 0.092 |
| **Tumor size (cm)** |  |  |  |  |
| No more than 3 | Reference |  | Reference |  |
| 3-5 | 1.700 (1.520,1.901) | <0.001 | 1.569 (1.398,1.762) | <0.001 |
| Larger than 5 | 2.262 (1.926,2.658) | <0.001 | 1.742 (1.428,2.125) | <0.001 |
| Unknown | 1.673 (1.357,2.062) | <0.001 | 1.415 (1.084,1.846) | 0.011 |
| **Ethnicity** |  |  |  |  |
| White | Reference |  | Reference |  |
| Black | 0.936 (0.801,1.094) | 0.405 | 0.958 (0.819,1.12) | 0.591 |
| Other | 0.748 (0.659,0.848) | <0.001 | 0.736 (0.646,0.839) | <0.001 |
| **Marital status** |  |  |  |  |
| Married | Reference |  | Reference |  |
| Unmarried | 1.111 (1.006,1.229) | 0.039 | 1.176 (1.06,1.305) | 0.002 |
| Unknown | 0.837 (0.628,1.115) | 0.224 | 0.901 (0.671,1.209) | 0.487 |
| **Chemotherapy** |  |  |  |  |
| Yes | Reference |  |  |  |
| No | 0.909 (0.818,1.009) | 0.072 | 1.070 (0.958,1.195) | 0.233 |
| **Treatment** |  |  |  |  |
| Cryoablation | Reference |  |  |  |
| RFA | 0.777 (0.592,1.02) | 0.069 | 0.887 (0.681,1.155) | 0.374 |

**Table 2: Predictors for competing risk after PSM**

| Characteristics | Univariate analysis |  | Multivariate analysis |  |
| --- | --- | --- | --- | --- |
|  | HR (95%CI) | P | HR (95%CI) | P |
| **Age (Years)** |  |  |  |  |
| 40-49 | Reference |  |  |  |
| 50-59 | 0.390 (0.134,1.130) | 0.083 | 0.340 (0.077,1.504) | 0.155 |
| 60-69 | 0.566 (0.189,1.695) | 0.309 | 0.862 (0.117,6.373) | 0.884 |
| 70-79 | 0.596 (0.195,1.822) | 0.364 | 1.387 (0.07,27.425) | 0.830 |
| **Gender** |  |  |  |  |
| Male | Reference |  |  |  |
| Female | 1.299 (0.864,1.953) | 0.208 |  |  |
| **Year of diagnosis** |  |  |  |  |
| 2004-2007 | Reference |  | Reference |  |
| 2008-2011 | 0.504 (0.279,0.913) | 0.024 | 0.264 (0.061,1.139) | 0.074 |
| 2012-2015 | 0.38 (0.163,0.888) | 0.025 | 0.163 (0.012,2.205) | 0.172 |
| **Tumor stage** |  |  |  |  |
| Localized | Reference |  | Reference |  |
| Regional | 0.515 (0.326,0.813) | 0.004 | 0.472 (0.228,0.978) | 0.043 |
| Unknown/Unstaged | 0.465 (0.125,1.724) | 0.252 | 2.239 (0.207,24.151) | 0.507 |
| **AJCC stage** |  |  |  |  |
| I | Reference |  | Reference |  |
| II | 1.313 (0.864,1.994) | 0.202 | 0.908 (0.513,1.605) | 0.739 |
| III | 3.576 (1.900,6.731) | <0.001 | 2.362 (0.856,6.517) | 0.097 |
| UNK stage | 0.691 (0.221,2.156) | 0.524 | 0.321 (0.032,3.207) | 0.333 |
| **Tumor size (cm)** |  |  |  |  |
| No more than 3 | Reference |  | Reference |  |
| 3-5 | 1.906 (1.124,3.23) | 0.017 | 1.757 (0.924,3.342) | 0.086 |
| Larger than 5 | 2.385 (1.409,4.036) | 0.001 | 1.414 (0.566,3.533) | 0.458 |
| Unknown | 0.914 (0.435,1.92) | 0.813 | 1.051 (0.38,2.906) | 0.923 |
| **Ethnicity** |  |  |  |  |
| White | Reference |  |  |  |
| Black | 1.608 (0.934,2.768) | 0.087 | 0.830 (0.283,2.434) | 0.734 |
| Other | 3.231 (1.45,7.198) | 0.004 | 0.922 (0.100,8.505) | 0.943 |
| **Marital status** |  |  |  |  |
| Married | Reference |  |  |  |
| Unmarried | 0.884 (0.582,1.343) | 0.563 |  |  |
| Unknown | 0.633 (0.253,1.582) | 0.327 |  |  |
| **Chemotherapy** |  |  |  |  |
| Yes | Reference |  |  |  |
| No | 1.149 (0.796,1.658) | 0.460 |  |  |
| **Treatment** |  |  |  |  |
| Cryoablation | Reference |  |  |  |
| RFA | 0.843 (0.643,1.105) | 0.215 |  |  |

**Figure 1:** The flowchart of patients inclusion


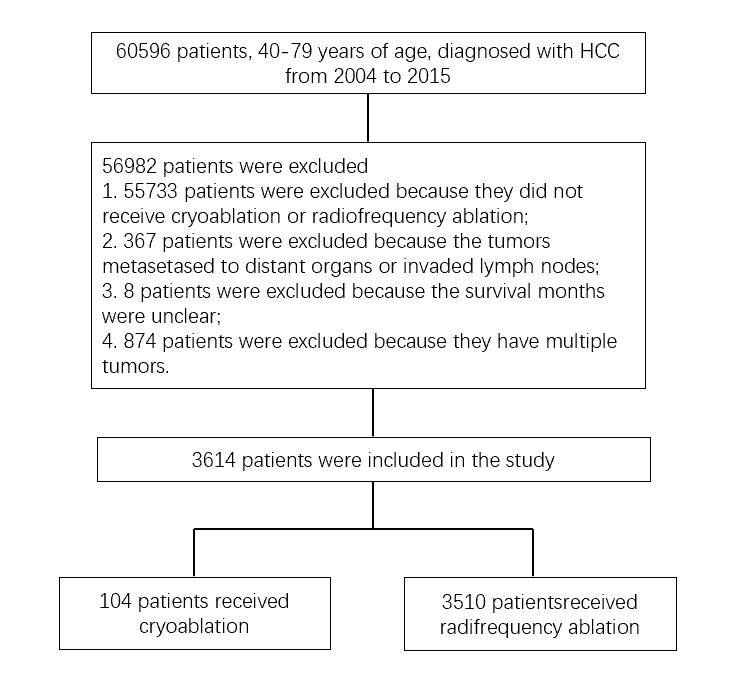


**Figure 2:** Kaplan-Meier curve of overall survival and cancer-specific survival of patients with cryoablation and radiofrequency ablation (RFA) before PSM. (A,B) patients with tumor size no more than 3 cm; (C,D) patients with tumor size no more than 5 cm; (E,F) patients with tumor size larger than 5 cm; (G,H) patients with AJCC I and II stage HCC


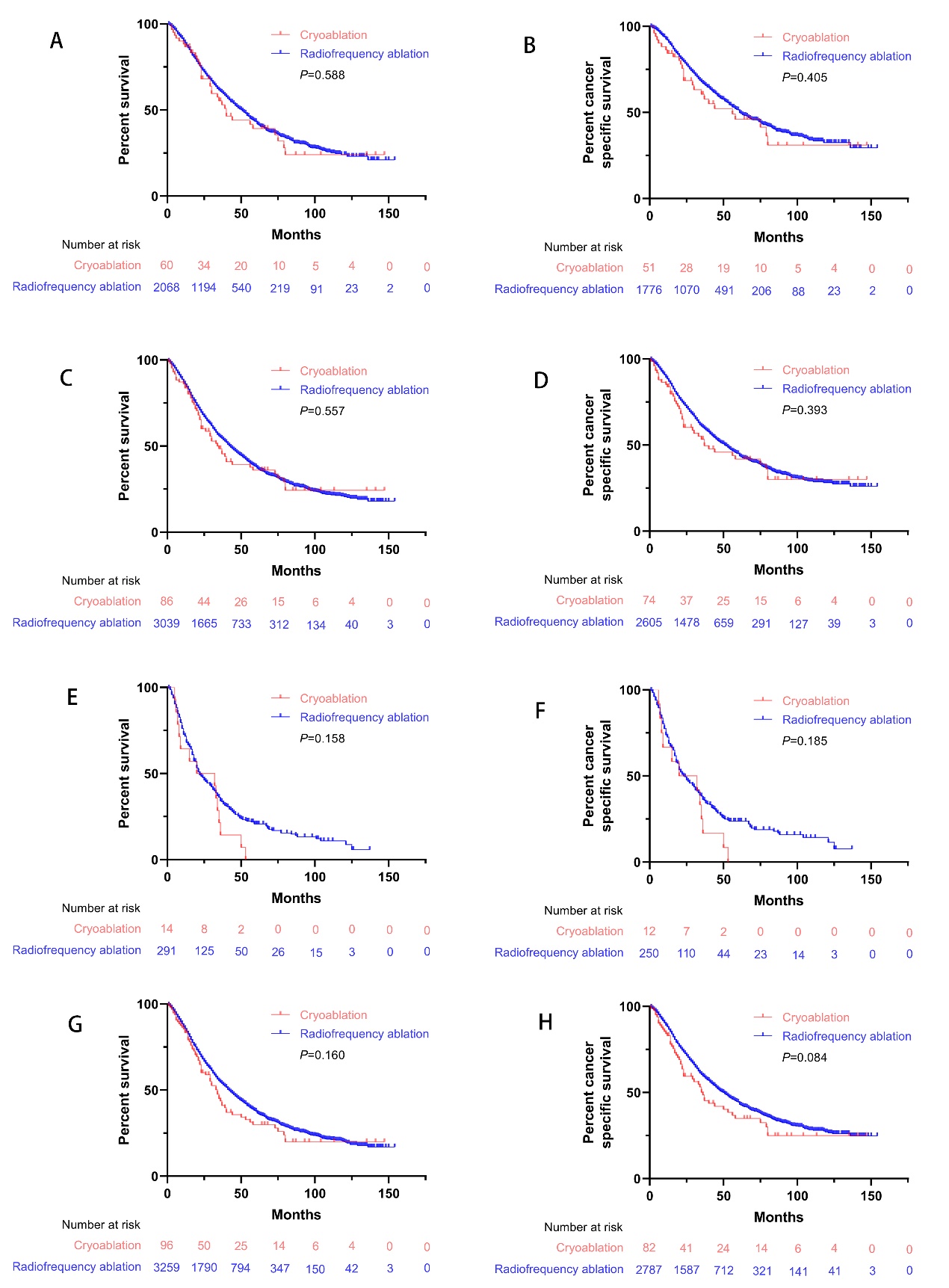

Supplement: Supplementary file 1 — Supplementary Material [file CAM4-10-3715-s001.docx]
